# Ubiquitous transient stagnant domain formation during thermal convection in a well-mixed two component fluid with large viscosity difference

**DOI:** 10.1038/s41598-017-13409-w

**Published:** 2017-10-11

**Authors:** Kazuya U. Kobayashi, Rei Kurita

**Affiliations:** Department of Physics, Tokyo Metropolitan University, 1-1 Minamioosawa, Hachioji-shi, Tokyo 192-0397 Japan

**Keywords:** Fluid dynamics, Nonlinear phenomena

## Abstract

The formation of a transient stagnant domain in the presence of thermal convection was previously reported near the sol-gel transition temperature of a gelatin solution. The transient stagnant domain is observed near a critical Rayleigh number where a "roll" pattern is usually stable. It is important to understand the origin of the transient stagnant domain formation since it induces a large deformation of convection patterns; the nature of the formation of the transient stagnant domain remains unclear. Here, we observe thermal convection using several different fluids and find that stagnant domain formation is ubiquitous in two component mixtures. In addition, we find that difference in viscosity between the two components is crucial for transient stagnant domain formation, more so than the concentration gradient induced by the temperature gradient.

## Introduction

Convection driven by the density difference between horizontal fluid layers when they are heated from below is called Rayleigh-Bénard convection (RBC). RBC is chiefly governed by the non-dimensional Rayleigh number, defined as:

1 $$Ra=\frac{g\alpha {\rm{\Delta }}T{H}^{3}}{\nu \kappa }$$

where *g*, *α*, Δ*T*, *H*, *v* and *κ* are acceleration due to gravity, the thermal expansion coefficient, the temperature difference across the fluid, sample height, kinematic viscosity and thermal diffusivity, respectively. RBC has been investigated extensively, both experimentally and theoretically, due to its relationship with pattern formation^1–4^, turbulent and chaotic dynamics^5–8^, and interest in the effect of inclined boundaries^9–12^. It is also of practical importance for many engineering applications and natural phenomena^4,13,14^.

It is known that RBC in a two component fluids is more complex than that in single component fluids. RBC in two component fluid systems is called double-diffusive convection. Double-diffusive convection in binary fluid mixtures has been investigated for its relation to hydrodynamic instability, structure formation, complex spatiotemporal behavior, turbulence, etc.^15–23^. In a binary fluid system, a concentration gradient is induced by the Ludwig-Soret effect in response to an externally imposed temperature gradient. Generally, the density of the mixture depends on the concentration; RBC is thus strongly affected by the concentration gradient. The effect of the concentration gradient is described by a parameter known as the “separation ratio” Ψ defined as:

2 $${\rm{\Psi }}=c\mathrm{(1}-c)\alpha {\beta }^{-1}{S}_{T}$$

where *c* is the weight fraction, *α* is the thermal expansion coefficient, and *β* is the mass expansion coefficient. *S*_*T*_ is the Soret coefficient, which is defined as follows:

3 $${S}_{T}=-\,\frac{1}{c\mathrm{(1}-c)}\frac{\nabla c}{\nabla T}.$$

If Ψ < 0, the denser component moves to the hot boundary, and the concentration gradient becomes stable. Therefore, the critical Rayleigh number is larger than that for a pure fluid system. An ethanol-water mixture is a typical example of this case. Meanwhile, when Ψ > 0, the denser component moves to the cold boundary. This means that the Ludwig-Soret effect enhances the buoyancy forces; the critical Rayleigh number becomes much smaller than that for a pure fluid system. A salt solution is a typical example of this case. Investigations of convection considering the separation ratio in two component systems include both experimental and theoretical works^15–23^.

Recently, we have studied thermal convection near the critical Rayleigh number *Ra* = 10^4^~10^5^ in a gelatin solution. We observe that a domain without flow is transiently formed in an upwelling near the upper surface and the mode of heat transfer in the fluid changes repeatedly between convection and conduction over time^24^. Furthermore, we also observed transient stagnant domain (TSD) in thermally driven convective flow in diluted Golden Syrup, which is not a viscoelastic fluid^25^. We also revealed that TSD formation is not caused by a local decrease in temperature. Although TSDs are crucial for the convection dynamics, the nature of TSD formation remains unclear.

In this Letter, we investigated convective dynamics in one component and two component systems using several different fluids. As a result, we determined that TSDs are observed in two component systems with a large viscosity difference between the components, more so than arising from the concentration dependence of the density. Our results suggest that dynamical asymmetry is a crucial factor determining fluid dynamics in binary mixtures.

## Results and Discussion

**Figure 1:**
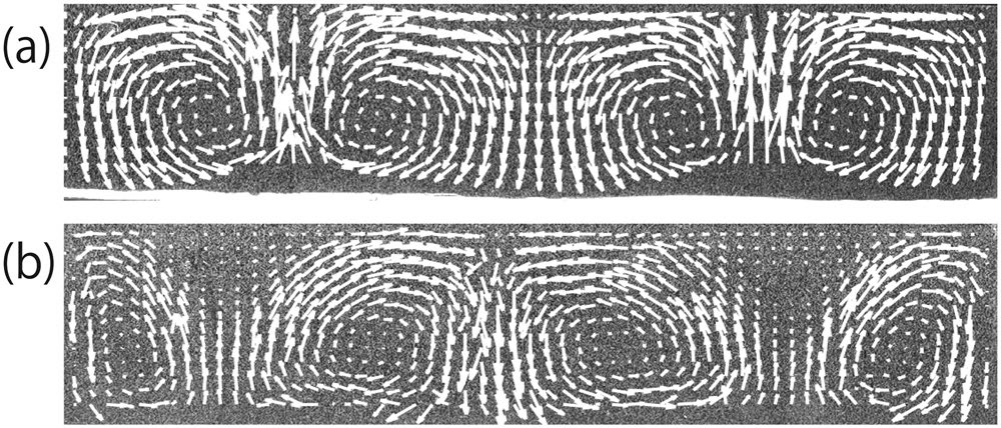
Snapshots of thermal convection in pure glycerol and 60 wt% glycerol (t = 34 min). The arrows indicate the flow. A regular “roll” pattern is formed in (**a**). The entire flow pattern is clearly changed by the TSD in (**b**). The temperature of the bottom surfaces is *T*_*b*_ = 90 °C in (**a**) and 48 °C in (**b**).

**Figure 2:**
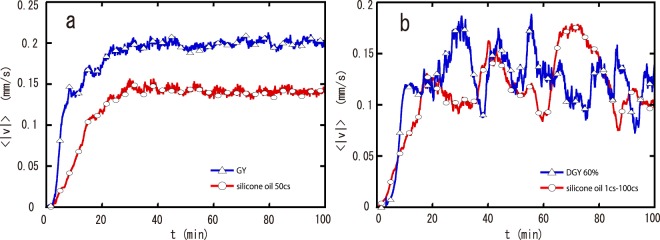
Time evolution of the average velocity $\langle |v|\rangle$ near the top surface in glycerol and silicone oil. (**a**) shows results for pure glycerol (blue line) and silicone oil 50cs (red line).The mean speed $\langle |v|\rangle$ is constant after the formation of convection. (**b**) shows results for 60% glycerol (blue line) and 1cs–100cs silicone oil mixtures (red line). $\langle |v|\rangle$ fluctuates several times; these fluctuations correspond to the formation of TSDs. The symbols are shown at every 200 th data point.

Firstly, we investigate the time evolution of thermal convection in pure glycerol (GY) and 60 wt% glycerol diluted with water (DGY). The velocity field is obtained by corrected particle image velocimetry (PIV). All the relevant physical properties are summarized in Table 1. Figure 1(a)(b) shows an image of the velocity field visualized using PIV at *t* = 34 min. Rayleigh numbers in our GY and DGY experiments are *Ra*_*GY*_ = 1.3 × 10^5^ and *Ra*_*DGY*_ = 1.2 × 10^5^, respectively. Figure 1(a) is a snapshot of the velocity field of GY. A “roll” convection is observed and is stable over time. Meanwhile, the convection in DGY is a “roll” pattern similar to that in GY; however, the roll pattern is not stable. A domain without flow (stagnant domain) is transiently formed near the top surface at the upwelling, shown in Fig. 1(b). The transient stagnant domain (TSD) subsequently induces a rearrangement of convection cells, and the roll pattern is reformed. This is the same as the convection dynamics observed in gelatin solution and diluted golden syrup, which were reported in^24,25^. Figure 2 shows the time evolution of the mean velocity $\langle |v|\rangle$ near the top surface. We compute $v(x,y,t)=\sqrt{{v}_{x}^{2}+{v}_{y}^{2}}$ for every PIV box. $\langle |v|\rangle$ is the mean of $|v|$ averaged over a region near the top surface *D*_*top*_, defined as ${D}_{top}=\{(x,y)|0 < x < W,H-3{\rm{\Delta }} < y < H\}$, where Δ is the size of the PIV box (1 mm). The blue line with triangle symbols in Fig. 2(a) shows the time evolution of $\langle |v|\rangle$ in GY. $\langle |v|\rangle$ is almost constant after the formation of a convective “roll” pattern. Meanwhile, $\langle |v|\rangle$ of the convection in DGY fluctuates, as seen from the blue line with triangle symbols in Fig. 2(b). Comparing it with the images, we confirm that the decrease in $\langle |v|\rangle$ corresponds to the formation of a TSD. This results suggests that TSD formation can be observed in a two component fluid.

**Table 1:** Physical properties of fluids^29–31^.

Fluid	Viscosity *η* (mPa s)	Density *ρ* (kg m^−3^)	*Pr*
water	0.47 (60 °C)-1.0 (20 °C)	998 (20 °C)	7.01 (20 °C)
glycerol	21 (90 °C)-1400 (20 °C)	1261 (20 °C)	12469 (20 °C)
silicone oil (1cs)	0.51 (50 °C)-0.82 (25 °C)	818 (25 °C)	16.4 (25 °C)
silicone oil (50cs)	31.2 (50 °C)-48 (25 °C)	960 (25 °C)	454.5 (25 °C)
silicone oil (100cs)	62.3 (50 °C)-96.5 (25 °C)	965 (25 °C)	909.1 (25 °C)
ethanol	0.83 (40 °C)-1.1 (20 °C)	789 (20 °C)	16.9 (20 °C)

**Figure 3:**
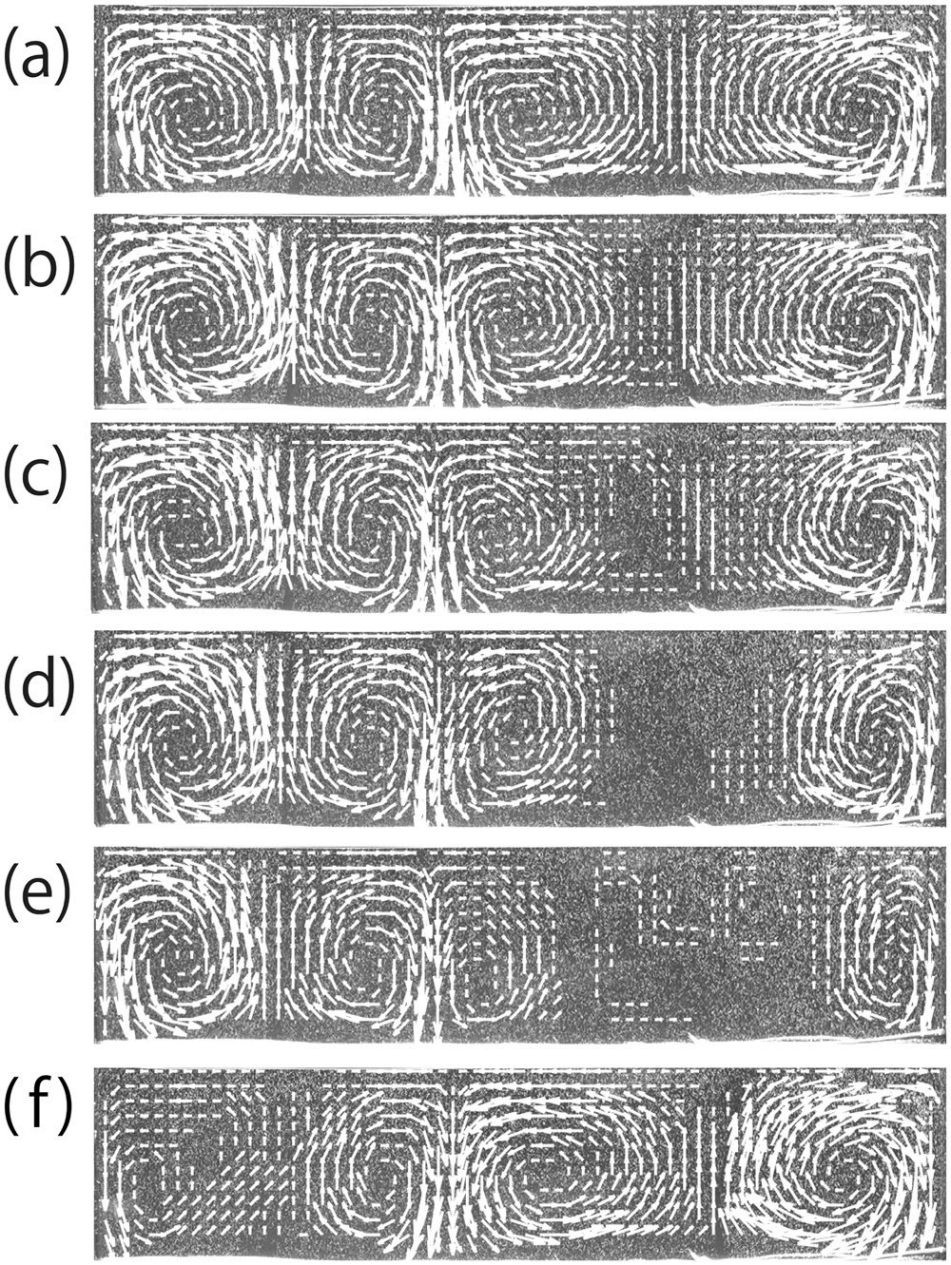
Pattern evolution during thermal convection in silicone oil, 1cs–100cs mixtures. (**a)** t = 56 min, (**b**) t = 57 min, (**c**) t = 58 min, (**d**) t = 60 min, (**e**) t = 62 min and (**f**) t = 80 min. A TSD is formed and the macroscopic flow of the entire system drastically changes. Because temperature-dependence on viscosity is very small, a stagnant domain with a pillar shape is formed in the whole upwelling. The temperature of the bottom surface is *T*_*b*_ = 48 °C.

To confirm this hypothesis, we investigate thermal convection using silicone oil, investigating pure 50cs oil and 1cs–100cs mixtures (4:6 mixture). We perform a convection experiment with Rayleigh number *Ra*_50_ = 9.6 × 10^4^ using pure 50cs silicone oil and *Ra*_1−100_ = 9.4 × 10^4^ for a mixture of 1cs and 100cs oils. Note that both *R*′_*a*_s are far below the chaotic regime^26^. Figure 3 show the time evolution of the velocity field in the 1cs–100cs mixture visualized by PIV at *t* = (a) 56 min, (b) 57 min, (c) 58 min, (d) 60 min, (e) 62 min, and (f) 80 min. A “roll” pattern is formed in the system (Fig. 3(a)). However, a TSD with a pillar shape is formed on the right side (Fig. 3(b) and (c)). Here, we note that the TSD is formed not only in the upwelling but also in the downwelling. The domain without flow expands over time (Fig. 3(d) and (e)). After these events, the convective flow gradually returns to the early stage (Fig. 3(f)). These results are consistent with our previous results^24,25^. Meanwhile, TSD formation was not observed in pure silicone oil. We show the time evolution of $\langle |v|\rangle$ in the pure silicone oil with viscosity 50cs and the mixture of 1cs–100cs, given in Fig. 2 (red lines with circle symbols). Figure 2(a) shows that the mean velocity $\langle |v|\rangle$ is constant; it is clear that a TSD is not formed in the one component system. On the other hand, $\langle |v|\rangle$ fluctuates several times in the mixture as seen from the red line with circles in Fig. 2(b). These fluctuations correspond to the formation of TSDs. We also find that TSD formation occurs repeatedly over 12 hours. Here, we note that the formation of TSDs is not sensitive to the mixing ratio. We also performed the experiment with the mixing ratio changed to 6:4 and then the TSD formation was observed. We also discuss the influence of Prandtl number *Pr* on the TSD formation. We calculated *Pr* for the 4:6 mixture of 1cs–100cs (*Pr*_1−100(4:6)_), the 6:4 mixture of 1cs–100cs (*Pr*_1−100(6:4)_), and the pure silicon oil of 50cs (*Pr*_50*cs*_). *Pr* is found to be *Pr*_1−100(4:6)_ = 368, *Pr*_1−100(6:4)_ = 218, and *Pr*_50*cs*_ = 272 by calculating with the viscosity values at the bottom surface. TSD formation could be observed in the mixture, however it does not appear in the pure silicon oil, even though the value of *Pr*_50*cs*_ is in between *Pr*_1−100(4:6)_ and *Pr*_1−100(6:4)_. Hence we conclude that the formation of TSD is not caused by the effects of *Pr*.

These results reflect additional evidence for TSD formation in two component mixtures. Note that the viscosities of silicone oils only depend weakly on temperature, while glycerol has a much more temperature sensitive viscosity. TSDs are formed near the top surface in fluids with a strong temperature dependence, such as in glycerol solutions, and a pillar-shaped TSD is found in silicone oil systems. Thus we find that TSD is less related with the temperature dependence on viscosity.

**Figure 4:**
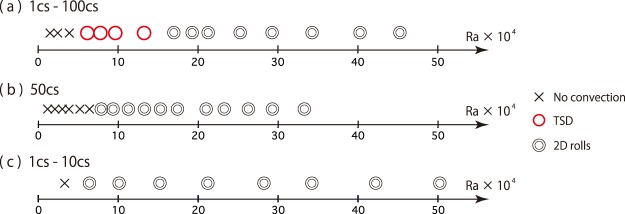
Regime diagram of the thermal convection for the case of 1cs–100cs (**a**), 50cs (**b**), and 1cs–10cs (**c**). cross: no convection, open circle: 2D-rolls with the formation of TSD, double open circle: 2D-rolls. (**a**) The convective regime changes from the TSD regime to the 2D-rolls regime as the Rayleigh number increases. (**b)**,(**c**) TSD regime does not exist and the state is in the 2D-rolls regime for the range experimentally measured.

Furthermore, we investigate Rayleigh number dependence on the flow dynamics. Figure 4 shows regime diagram of thermal convection in 1cs–100cs, 50cs, and 1cs–10cs. In the two-component system of 1cs–100cs, the convective regime changes from the TSD regime to the 2D-roll regime as the Rayleigh number increases. In case of the one-component system (50cs), TSD regime does not exist and the state is in the 2D-rolls regime within the experimental range. Moreover, in the two-component system of 1cs–10cs, where the viscosity difference is small, we also observe no TSD regime within the experimental range.

**Figure 5:**
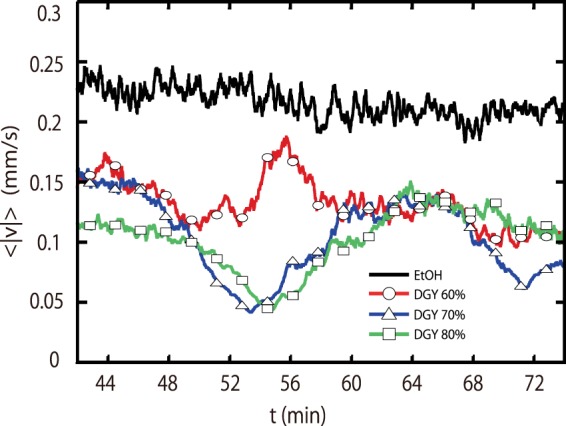
Time evolution during thermal convection in 60 wt% (red line), 70 wt% (blue line), 80 wt% glycerol (green line), and 30 wt% ethanol (black line). TSD formation is observed in glycerol solutions with a positive *S*_*T*_, almost 0, and negative *S*_*T*_. Meanwhile we could not see TSD formation in the ethanol solution. The symbols are shown at every 100th data point.

Next, we show that TSD formation is not related to the Ludwig-Soret effect. As we described above, the convection dynamics in mixtures is critically affected by the Ludwig-Soret effect. We thus investigate TSD formation in mixtures where the Ludwig-Soret effect can be negligible. According to ref.^27^, *S*_*T*_ depends on the weight fraction *c*; the sign of *S*_*T*_ can also change. That is, we can set *S*_*T*_ close to 0 by changing *c*. Thus we prepare mixtures with *S*_*T*_ ~ 0 and investigate their convection dynamics. We show the mixtures we used, their *S*_*T*_, whether we observe TSDs, and the number of experiments carried out, *N*_*exp*_ in Table 2. The value of *S*_*T*_ in our experiment is much smaller than that in the experiment looking at double diffusive convection (~7.2 × 10^−3^ for ethanol 8 wt% solution). Figure 5 shows $\langle |v|\rangle$ in 60 wt% glycerol, 70 wt% glycerol, 80 wt% glycerol, and 30 wt% ethanol. The sign of *S*_*T*_ is slightly positive for 60 wt% glycerol, while it is slightly negative for 80 wt% glycerol. Furthermore, *S*_*T*_ is almost 0 in 70 wt% glycerol, which means that a concentration gradient is not induced by the temperature gradient. We nevertheless find that TSD formation is observed for convection in 60 wt%, 70 wt%, and 80 wt% glycerol. This result suggests that TSD formation is not related to the Ludwig-Soret effect. Meanwhile, TSDs are not formed in 30 wt% ethanol, where *S*_*T*_ is also negligible. This means that TSDs are not formed unconditionally just because a mixture has two components.

**Table 2:** Physical properties of the mixtures we used, whether TSDs are observed or not, and the number of the experiments carried out.

Mixture	*v*_*R*_	*S*_*T*_ (K^−1^)	TSD	*N*_*exp*_
glycerol (60 wt%) + water	1117	5.5 × 10^−4^	$\circ$	10
glycerol (70 wt%) + water	1117	<10^−4^	$\circ$	5
glycerol (80 wt%) + water	1117	−8.7 × 10^−4^	$\circ$	5
silicone oil (1 + 10)	10	NA^a^	×	3
silicone oil (1 + 50)	50	NA	$\circ$	3
silicone oil (1 + 100)	100	NA	$\circ$	10
silicone oil (50 + 100)	2	NA	×	3
ethanol (30 wt%) + water	1.518	<10^−4^	×	10
ethanol (35 wt%) + water	1.518	−1.39 × 10^−3^	×	3

*ν*_*R*_ for the glycerol solutions is calculated using the viscosity at *T* = 20 °C. ^a^NA, not available.

We also examined the effect of the temperature dependence of interfacial tension (Marangoni effect). Marangoni effect cannot be neglected in our experimental setup because the top boundary is a free surface. Thus we perform the additional experiment with experimental setup where the top boundary is uniformly cooled. In this condition, we confirm the TSD formation. This result suggests that the TSD formation is not related to the Marangoni effect.

We now focus on the viscosity difference between the two components of the mixture. We define the ratio of viscosities *v*_*R*_ as ${\nu }_{R}={\nu }_{l}/{\nu }_{s}$, where *v*_*l*_ and *v*_*s*_ are the viscosities of the more viscous and less viscous component, respectively. We show *v*_*R*_ for each mixture in Table 2. We find that TSD formation occurs when *v*_*R*_ is large, and not in the convection of ethanol-water mixtures and mixtures of silicone oil with *v*_*R*_ ≤ 10. These results are consistent with our previous findings in diluted golden syrup^25^. The viscosity of the golden syrup is also much larger than that of water. Therefore, dynamical asymmetry between the two components in the mixture seems crucial for TSD formation. Here, we introduce the key phenomenon which is related to the formation of TSD^32^. Even in the well mixed binary mixture, a fluctuation of the concentration exists. When viscosity strongly depends on the concentration, a spatial distribution of viscosity is induced in the mixture. The viscosity distribution can be coupled with simple shear, that is, the inhomogeneity of the viscosity distribution can be enhanced by simple shear. Since shear stress is present in the convection, it is thought that domains of large viscosities could be formed by the fluctuation of the concentration and the convection flow. However the convection is intricately coupled with velocity field, concentration field, density field, viscosity, and temperature field. Therefore, to elucidate the TSD formation a simulation study and the stability analysis are needed.

## Conclusion

To summarize, we experimentally studied TSD formation using several different fluids. We found that a TSD is formed in mixtures of fluids with a large viscosity difference like 60% glycerol in water and a mixture of two silicone oils, 1cs and 100cs. However, a TSD is not formed in one component fluids like pure glycerol and silicone oil 50cs. We also show that TSD formation is not as closely related to the Ludwig-Soret effect as previously believed. We conclude that it is, in fact, the viscosity difference between the two components of the fluid mixture that is crucial for TSD formation. Although, how the viscosity difference plays a role for the TSD formation is unclear. In the future work we plan to clarify the relation between viscosity ratio and the TSD formation by the stability analysis and numerical simulation studies. We hope our results stimulate further discussion regarding the physics of convection and non-equilibrium phenomena with dynamical asymmetry.

## Methods

We performed the experiment with glass sample chambers with internal dimensions (H, L, W) = (12 mm, 56 mm, 2.4 mm). We also performed the same experiments using a long sample chamber (H, L, W) = (12 mm, 136 mm, 2.4 mm) and obtained the same results. Thus we only show the results for (H, L, W) = (12 mm, 56 mm, 2.4 mm) in this paper. Our sample chambers are quasi two dimensional Hele-Shaw cells for accurate analysis of flow and structure in the vortices^12^. We controlled the temperature of the bottom surface using a temperature controller (S100 Blast Co.) and kept the top surface free. The room temperature is set to 22.1 by a standard air conditioner. We used glycerol 99% manufactured by SIGMA-ALDRICH and ethanol 99% purchased from Wako Pure Chemical Industries Co., Ltd. We also used silicone oils (KF-96L-1cs, KF-96-50cs, KF-96-100cs) manufactured by Shin-Etsu Chemical Co., Ltd. To prepare a well-mixed solution, the two component mixture was thoroughly stirred with a stirrer and was mixed further in an ultrasound bath for more than 30 minutes. We used aluminum powder and polystyrene (PS) latex for the visualization of the velocity field, manufactured by Wako Pure Chemical Industries Co., Ltd. We then used particle image velocimetry (PIV) to carry out a quantitative analysis of the flow^28^. Images visualized by aluminum powder and PS latex were recorded with a digital camera (Model HC-V520M, Panasonic Co.) at 1 s intervals. More details on the experimental method are given in our previous articles^12,24,25^.
