# Publisher Correction: Ubiquitous transient stagnant domain formation during thermal convection in a well-mixed two component fluid with large viscosity difference

**DOI:** 10.1038/s41598-019-45274-0

**Published:** 2019-06-13

**Authors:** Kazuya U. Kobayashi, Rei Kurita

**Affiliations:** Department of Physics, Tokyo Metropolitan University, 1-1 Minamioosawa, Hachioji-shi, Tokyo, 192-0397 Japan

Correction to: *Scientific Reports* 10.1038/s41598-017-13409-w, published online 11 October 2017

The publication date was omitted from the original PDF version of this Article. This error has now been corrected in the PDF; the HTML version of the paper was correct from the time of publication.

